# Measures of Heart Rate Variability in 24-h ECGs Depend on Age but Not Gender of Healthy Children

**DOI:** 10.3389/fphys.2017.00311

**Published:** 2017-05-18

**Authors:** Waldemar Bobkowski, Magdalena E. Stefaniak, Tomasz Krauze, Katarzyna Gendera, Andrzej Wykretowicz, Jaroslaw Piskorski, Przemyslaw Guzik

**Affiliations:** ^1^Department of Pediatric Cardiology and Nephrology, Poznan University of Medical SciencesPoznan, Poland; ^2^Department of Cardiology- Intensive Therapy and Internal Diseases, Poznan University of Medical SciencesPoznan, Poland; ^3^Institute of Physics, University of Zielona GoraZielona Gora, Poland; ^4^Faculty of Medicine and Health Sciences, University of Zielona GoraZielona Gora, Poland

**Keywords:** heart rate variability, healthy children, poincare plots, lomb-scargle periodograms, short-term HRV, long-term HRV, holter electrocardiograms

## Abstract

Many methods computing heart rate variability (HRV) have been applied in studies in children. Not all of these methods have a comprehensive physiological interpretation, and not all of studies are in agreement with the Task Force Standards on HRV from 1996, and the New Joint Position Statement on the advances of HRV from 2015. The study aim was to analyse HRV in the 24-h ECGs of healthy children by the Poincare plots and Lomb-Scargle periodograms, and to follow proper HRV recommendations. Additionally, we investigated the associations between age, children's sex and measured HRV indices. One hundred healthy children, aged 3–18 underwent 24-h ECG Holter monitoring. HRV was analyzed by the Poincaré plots and spectral by Lomb-Scargle periodograms of RR intervals. The Mann-Whitney test was used to compare sex differences in HRV, the van Elteren's test was used to correct for the age-gender interaction, and non-parametric Spearman correlation was applied to analyse the association between age and HRV indices. None of the HRV measures differed significantly between boys and girls. None of the HRV indices was modified by the age-gender interaction. There were statistically significant associations of age with measures of ultra-low (rho = 0.42; *p* < 0.0001), very low (rho = 0.35; *p* = 00004) and low (rho = 0.30; *p* = 0.0028) frequency powers, the ratio of the low to high frequency power (rho = 0.38; *p* = 0.0001), indices of long-term (SD2; rho = 0.37; *p* = 0.0002) and total (SDNN; rho = 0.33; *p* = 0.0008) HRV, and the contribution of the long-term HRV to total HRV (CL; rho = 0.32; *p* = 0.0012). In general, HRV parameters derived from the analyses of Poincaré plots and Lomb-Scargle periodograms appear not to be affected by gender, however, most of them increase with age in the 24-h ECG recordings in healthy children.

## Introduction

Heart rate variability (HRV) is the physiological variation in the duration of cardiac cycles. HRV is analyzed by many different mathematical algorithms (Task Force of the European Society of Cardiology the North American Society of Pacing Electrophysiology, 1996; Sassi et al., 2015). For many years HRV has been calculated with the use of stand-alone computers, nowadays it can easily be computed by mobile and wearable devices (Heathers, 2013; Guzik and Malik, 2016). So far HRV analysis has been applied in research, for both clinical and non-clinical purposes (Task Force of the European Society of Cardiology the North American Society of Pacing Electrophysiology, 1996; Sassi et al., 2015). Some examples of HRV applications are prediction of the risk of premature mortality after myocardial infarction (Bigger et al., 1992; La Rovere et al., 1998; Stein and Reddy, 2005; Guzik et al., 2012) or development of congestive heart failure (Patel et al., 2017), diagnosis of autonomic dysfunction in diabetes (Akinci et al., 1993; Schein et al., 2009), non-invasive estimation of the autonomic modulation of the cardiovascular system during stress (Srivastana, 2014), relaxation (Quintana and Heathers, 2014) or the assessment of the effects of physical training on fitness level (Bernardi et al., 2001; Makivić et al., 2013; Sanchez-Gonzalez et al., 2015). All of these are the reasons why the interest in HRV is growing both in clinical and physiological studies.

There are many mathematical methods applied to compute HRV—they may be grouped into statistical, spectral, graphical, non-linear, complexity, or information based (Task Force of the European Society of Cardiology the North American Society of Pacing Electrophysiology, 1996; Sassi et al., 2015). Among all of the methods and parameters which are in use only some are really comprehensive, have a good physiological interpretation or are perhaps better suited for the analysis of cardiovascular time series of RR intervals. The most classic parameters such as the mean duration of the RR interval or standard deviation of NN intervals (normal-to-normal RR intervals) (SDNN) are examples of HRV measures which are widely understood (Task Force of the European Society of Cardiology the North American Society of Pacing Electrophysiology, 1996; Sassi et al., 2015). Some other parameters like SD1 and SD2 (see Methods for their explanation) from the Poincare plot analysis of RR intervals, have excellent physiological explanation (Brennan et al., 2001; Guzik et al., 2010; Sassi et al., 2015). SD1 depends on instant changes of each pair of heart beats, i.e., two consecutive RR intervals, and thus describes the shortest possible HRV (its physiological interpretation is identical with rMSSD, i.e., the root mean square of the successive differences between RR intervals, and in fact both are rescaled by a constant, i.e., the square root of 2) (Brennan et al., 2001; Guzik et al., 2007, 2010; Sanchez-Gonzalez et al., 2015; Sassi et al., 2015). On the other hand, SD2 depends on the changes of the mean duration of each pair of heart beats, alters much more slowly and thus is considered as an index of the long-term HRV (Brennan et al., 2001; Guzik et al., 2010; Sassi et al., 2015). Finally, there are several methods applied for the spectral analysis of HRV, for example: Discrete Fourier Transform by Fast Fourier Transformation, autoregressive models, discrete wavelet transform or Lomb-Scargle periodograms all of which are believed to retrieve information from the autonomic regulation of heart rate by reflecting vagal tone and sympatho-vagal balance (Lomb, 1976; Bigger et al., 1992; Moody, 1993; Parati et al., 1995; Task Force of the European Society of Cardiology the North American Society of Pacing Electrophysiology, 1996; Eckberg, 1997; Bernardi et al., 2001; Verlinde et al., 2001; Pichon et al., 2006; Rajendra Acharya et al., 2006; Piskorski et al., 2010; Cysarz et al., 2011; Heathers, 2014; Sassi et al., 2015). Whereas the most commonly applied Fast Fourier Transformation requires resampling of the RR interval time series to get all the samples evenly distributed, the Lomb-Scargle periodogram does not need this. The Lomb-Scargle method can be reliably and directly applied to the irregularly sampled data (Lomb, 1976; Moody, 1993; Laguna et al., 1998; Piskorski et al., 2010). According to Moody (1993), only the Lomb-Scargle method, compared with Fast Fourier Transform and autoregressive models spectra, produces robust power spectrum density estimates in the presence of irregularly sampled signal such as an instantaneous heart rate, which can be additionally entwined from time to time by artifacts or ectopic beats. This method avoids all mathematical and technical problems of resampling and replacement of outliers, and introduces few drawbacks of its own. Finally, Moody recommends the Lomb-Scargle method as a method of choice for the spectral analysis of HRV (Moody, 1993). On the other hand it could be argued that, in general, being a least squares fit to a model, Lomb-Scargle periodogram strongly reflects the assumptions of the model. Additionally, in this method the concept of phase is lost as well as the ability to study the effect of filtering on the signal. However, according to Laguna et al. (1998) these drawbacks are not limiting in the case of the RR intervals time series and HRV.

HRV has been studied for clinical and physiological purposes in children for many years, and the relationship of HRV with gender and age is one of such examples. Despite there being many studies on this topic, data on the effects of gender and age on HRV in children are sparse (Korkushko et al., 1991; Clairambault et al., 1992; Finley and Nugent, 1995; Goto et al., 1997; Umetani et al., 1998; Silvetti et al., 2001; Rękawek et al., 2003; Lenard et al., 2004; Fukuba et al., 2009; Cysarz et al., 2011; Michels et al., 2013; Seppälä et al., 2014; Gasior et al., 2015; Jarrin et al., 2015; Sharma et al., 2015). There are many technical and methodological limitations of most of the studies: using ECG or non-ECG signals (e.g., from chest heart rate monitors); whether identification of the beat types and thus potential inclusion of non-sinus beats was present; whether replacing interpolation of the missing RR intervals was performed; different sampling frequency of ECG from 100 to 1,000 Hz or higher; recording the signals at various conditions, for different length of time from 1 min to 24 h, with averaging the 1-h segments separately for day and night or no averaging for the whole 24-h recording; using very small samples of studied children; enrolment of children in either a narrow range of or even exact age, e.g., 10 years old, or kids in a very wide range of age, sometimes with addition of young adults up to 22 years old; using different criteria for the healthy status; applying different mathematical algorithms for the same HRV parameters; using either RR intervals or heart rate; normalizing RR intervals to heart rate, etc. As a result—in spite of a huge number of publications there are many disparities and no uniform conclusions can be made which complicates and limits the practical use of HRV among pediatricians. Whereas some studies report on the existence of sex differences in HRV parameters, some others deny it. Similar conflicting data are on the relation of HRV with age—whereas some reports show age dependence, others either do not analyse this issue at all or do not confirm its existence.

As already mentioned, both the Poincare plots and the analysis of Lomb-Scargle periodograms of RR intervals have good physiological interpretations, and using their algorithms for the irregularly sampled RR intervals does not break any mathematical assumptions and does not introduce any intermediate, artificial signals. Both methods can be applied to the 24-h ECG recordings. However, to the best of our knowledge none of these methods have been applied to the 24-h ECGs collected in healthy children. For this reason, the primary, practical aim of this study was to perform the analysis of HRV in the 24-h ECGs by the Poincare plots and Lomb-Scargle periodograms in healthy children, and our intention was to exactly follow the requirements of the Task Force Standards on HRV measurement published in 1996 and the new joint position statement on the advances of HRV from 2015 (Task Force of the European Society of Cardiology the North American Society of Pacing Electrophysiology, 1996; Sassi et al., 2015, respectively). Therefore, the secondary aim of our study was to analyse the association of sex and age in healthy children with the applied measures of HRV in 24-h ECG Holter recordings.

## Materials and methods

We selected 100 healthy children aged 3–18 years from 1.6 thousand patients visiting our Outpatient Cardiac Paediatric Clinic in the years 2011-2014. The typical reasons for visits of these children were the differential diagnosis of heart murmurs, fainting, palpitations, chest pain and impaired exercise tolerance. The primary selection criterion was the availability of information from a complete cardiac clinical assessment based on the medical history, physical examination, standard biochemical analyses, 12-lead resting ECG, 24-h Holter monitoring, and echocardiography. From these patients we only included children with no acute or chronic disease, not taking any medication, with no abnormalities in the physical examination with the exception of the presence of an innocent (revealed in the course of diagnostic approach) heart murmur, normal resting 12-lead ECG (normal PQ, QRS and QT duration, no signs of any cardiac chamber hypertrophy, dilation or strain, no ECG signs of any typical channelopathy), no serious ventricular and supraventricular arrhythmias (i.e., no tachycardias), no pathological bradycardia due to sinus node or atrioventricular node dysfunction in 24-h ECG Holter monitoring, no structural or functional abnormality in the resting transthoracic echocardiography, and with normal results of standard biochemical analyses. Standard 12-lead ECG was recorded in supine position at rest, and all measurements of PQ, QRS, and QT were done manually. For the calculation of the corrected QT (QTc) we used the Bazett's formula (Bazett, 1920) Additionally, we did not include children who suffered from any serious infection at least 4 weeks before visiting the Outpatient Clinic as well as excluding all subjects participating in endurance sports. Consequently, all children accepted for this study were healthy.

Parents of all children as well as children of at least 7 years old themselves gave their informed consent for the enrolment to the study. We received permission from the local University Bioethical Committee.

## Methods

### 24-h ECG holter recording

Children underwent an ambulatory 24-h three-channel ECG Holter recording (Schiller Medilog Darwin, Schiller, Switzerland and since 2014 Schiller Medilog Darwin 2, Schiller, Switzerland) with 1,000 Hz sampling frequency of ECG. Initially, all recordings were automatically analyzed, then visually scanned and inspected to determine whether all beats were classified appropriately. If necessary, all misclassified beats and artifacts were manually corrected. The analyzed recordings were then exported to text files containing the duration of each cardiac cycle and an annotation of the beat type (sinus, supraventricular, ventricular or a technical artifact).

### Heart rate and heart rate variability

According to recommendations on HRV measurement (Task Force of the European Society of Cardiology the North American Society of Pacing Electrophysiology, 1996), only RR intervals of sinus origin were used for the quantification of the mean RR interval and HRV parameters. The following HRV parameters were quantified (Task Force of the European Society of Cardiology the North American Society of Pacing Electrophysiology, 1996; Brennan et al., 2001; Guzik et al., 2010; Piskorski and Guzik, 2011; Sassi et al., 2015):

- SDNN—standard deviation of normal-to-normal RR intervals as a measure of total HRV;
- SD1—standard deviation measuring the dispersion of points in the Poincare plot of RR intervals across the identity line. It is a measure of short-term HRV arising from instant beat-to-beat changes in the duration of RR intervals (SD1 is a rescaled root mean square of the successive differences—rMSSD—of RR intervals with identical physiological interpretation). Importantly, the Poincare plot analysis used for the quantification of HRV parameters presented here is defined in the RR_n_ − RR_n+1_ space—meaning that each point in the Poincare plot is described by the two neighboring RR intervals: the current RR interval (RR_n_) and the next RR interval (RR_n+1_);
- SD2—standard deviation measuring the dispersion of points in the Poincare plots of RR intervals along the identity line as a measure of the long-term HRV arising from the changes of the mean of two consecutive RR intervals;
- CL—the contribution of the long-term HRV to the total HRV that is a percentage of the long-term variance (SD2^2^) of RR intervals to doubled total variance of RR intervals (SDNN^2^).

$$\text{CL}=({\text{SD}2}^{2})/(2\times {\text{SDNN}}^{2})[\%].$$

The doubled total variance of RR intervals is a sum of the short-term variance (SD1^2^) and the long-term variance (SD2^2^):

$$\text{SD}1^{2}+\text{SD}2^{2}=2\times {\text{SDNN}}_{,}^{2}$$

Therefore, the contribution of the long-term variance of RR intervals, i.e., CL, to the total HRV is always opposite to the short-term (CS) contribution to the total HRV, and both sum up to 100%:

$$\begin{array}{l} \text{ CS}=(\text{SD}1^{2})/(2\times {\text{SDNN}}^{2})[\%]\\ \text{CS}+\text{CL}=100\%\\ \text{ CL}=100\%-\text{CS} \end{array}$$

To avoid showing just mathematical rather than physiological associations, we only present the results for CL, without CS. The interpretation of CL is how much total HRV comes from the long-term variability of RR intervals, and that the remaining part origins from CS (i.e., short-term HRV). Because this is a relative measure with total HRV represented by 100%, the CL is self-explanatory—it helps to understand that as CL cannot reach the value of either 0 or 100% it is instantly obvious that the remaining part of total HRV must come from the short-term HRV.

For the spectral analysis the method of Lomb-Scargle periodograms was applied (Lomb, 1976; Moody, 1993; Laguna et al., 1998; Piskorski et al., 2010), and the following parameters were calculated (Task Force of the European Society of Cardiology the North American Society of Pacing Electrophysiology, 1996; Sassi et al., 2015):

- ULF—the power of ultra-low frequency (0.00–0.0033 Hz) of RR intervals;
- VLF—the power of very low frequency (0.0033–0.04 Hz) of RR intervals;
- LF—the power of low frequency (0.04–0.15 Hz) of RR intervals;
- HF—the power of high frequency (0.15–0.4 Hz) of RR intervals;
- LF/HF—the ratio of the powers of LF to HF;

We have deliberately omitted the HF and LF in normalized units since there is a direct mathematical relationship between the two (Task Force of the European Society of Cardiology the North American Society of Pacing Electrophysiology, 1996; Eckberg, 1997; Burr, 2007; Piskorski et al., 2010; Heathers, 2014; Sanchez-Gonzalez et al., 2015; Sassi et al., 2015). Therefore, information redundancy is inherent in the normalized spectral HRV measures with respect to each other (LF_nu_ = 1 − HF_nu_) and also with respect to the LF/HF ratio (LF/HF = LF_nu_/(1 − LF_nu_) (Burr, 2007; Piskorski et al., 2010; Heathers, 2014; Sanchez-Gonzalez et al., 2015). Therefore, the physiological information derived from LF/HF, LF_nu_, and HF_nu_ is the same. We did not focus on the total power of RR intervals from spectral analysis either, although it was calculated together with LF and HF with the Lomb-Scargle periodograms. The total spectral power of RR intervals is always an approximation of total variability and it is never more accurate than SDNN. Therefore, to avoid another redundancy, we used SDNN as the only measure of total HRV.

In-house software written in Python (Python Foundation, Ipswich, MA, USA) was applied for all analyses of RR intervals.

### Statistical analysis

The Shapiro-Wilk test showed that data distribution was non-Gaussian, and hence they were described as mean, standard deviation (SD), median and the 25th and 75th percentile. The comparison of HRV parameters between girls and boys was made with the non-parametric Mann-Whitney test. To address the issue of potential interaction between age and children's sex in the Man-Whitney test we controlled for the age factor by carrying out the stratified van Elteren's test, which is an extension of the Man-Whitney test (Kawaguchi and Koch, 2010, 2015). All children were divided into three age groups, i.e., 3–6 years old corresponding to early childhood (Group 1); 7–12 years old corresponding to preadolescence (Group 2); and 13–18 years old, i.e., adolescence (Group 3). This division also corresponds to the school system in Poland according to the children's age, i.e., preschool period for children <7 years old; primary school for children between 7 and 12 years old, and the middle and high school for children >12 years old. The comparison between age groups was made with the use of the non-parametric Kruskal-Wallis test with post-tests. Since the age group is an ordered qualitative factor, the Jonckheere-Terpstra trend test was applied to evaluate the hypothesis that the medians of the analyzed parameters are ordered either in an increasing or decreasing direction according to the order of the qualitative factor (Bewick et al., 2004; Sheskin, 2011). Additionally, for the analysis of the association between age and HRV parameters the non-parametric Spearman correlation was applied. Statistical analyses were carried out with Prism for Windows (GraphPad, USA) and Medcalc for Windows (Medcalc, Belgium). Only *p* < 0.05 were considered significant.

## Results

### Clinical characteristics and results of HRV analysis according to gender

There were 51 girls and 49 boys—their clinical characteristics and the results of the comparison by the Mann-Whitney test are shown in Table 1. Girls were significantly older (two-years difference in median age), had shorter duration of QRS (10 ms difference in median duration of QRS) and longer duration of QTc (7 ms difference in median duration of QTc) than boys. No other clinical parameters, including all HRV indices, were different between girls and boys.

**Table 1:** **Clinical characteristics and results of HRV analysis as well as comparison between girls and boys by the Mann-Whitney test, and the van Elteren's test for the analysis of the influence of the interaction between age and sex on the analyzed parameters**.

	**Girls *N* = 51**					**Boys *N* = 49**					**M-W test *p*-value**	**van Elteren's test**
	**Mean**	***SD***	**Median**	**25th p**.	**75th p**.	**Mean**	***SD***	**Median**	**25th p**.	**75th p**.		
Age [years]	13.9	3.7	15.0	13.0	16.0	12.3	3.9	13.0	9.8	15.0	0.0025	–
PQ [ms]	129.0	14.7	130.0	120.0	140.0	129.9	17.8	130.0	120.0	142.8	0.6125	0.5654
QRS [ms]	83.8	8.6	80.0	80.0	90.0	87.2	9.0	90.0	80.0	91.0	0.0422	0.0001
QTc [ms]	398.1	16.7	397.0	387.3	411.5	387.6	21.7	390.0	370.8	400.0	0.0288	0.01394
Supraventricular beats in 24-h ECG [%]	0.000235	0.0009293	0.0	0.0	0.0	0.00173	0.006480	0.0	0.0	0.0	0.3575	0.05593
Ventricular beats in 24-h ECG [%]	0.000176	0.0005179	0.0	0.0	0.0	0.205	1.4248	0.0	0.0	0.0	0.9589	0.1525
Duration of ECG recording [min]	1287.157	119.5533	1297.832	1202.0	1385.2	1313.218	81.7335	1302.376	1259.1	1370.6	0.5372	0.6075
Mean RR [ms]	736.2	73.0	736.5	688.3	783.1	744.9	87.2	758.6	679.2	805.6	0.8930	0.0242
SDNN [ms]	159.0	43.1	161.5	132.6	190.2	158.0	39.9	157.1	126.1	190.3	0.4139	0.5090
ULF [ms^2^]	21732.3	12472.1	19582.6	12536.1	30669.4	19821.3	10731.4	18062.3	10912.7	26070.9	0.2262	0.8763
VLF [ms^2^]	3501.0	1648.8	3396.7	2095.2	4541.7	3928.5	2241.4	3279.0	2256.6	5845.1	0.8442	0.1359
LF [ms^2^]	1925.6	1164.9	1765.5	1072.6	2399.0	2097.8	1107.5	1723.1	1319.4	2906.1	0.8066	0.0813
HF [ms^2^]	1844.4	2077.5	1476.4	599.4	2139.8	1957.5	1734.5	1376.0	760.8	2286.6	0.3466	0.4395
LF/HF []	1.5	0.7	1.4	1.1	1.8	1.5	0.7	1.3	0.9	2.1	0.2236	0.7865
SD1 [ms]	47.7	25.6	43.9	28.5	56.5	50.3	23.6	48.2	32.3	60.0	0.6766	0.4474
SD2 [ms]	218.3	58.5	223.7	179.3	263.7	216.1	54.5	210.7	172.2	261.7	0.7433	0.5152
CL [%]	95.1	4.3	96.5	94.2	97.6	94.4	4.4	95.9	93.3	97.2	0.1030	0.5354

### Clinical characteristics and results of HRV analysis according to the interaction of gender and age

The Elteren's test revealed that the interaction between age and gender of the studied children might have some effect on the duration of QRS, QTc, and mean RR interval. None of the HRV parameters was influenced by the interaction between children's sex and age.

### Clinical characteristics and results of HRV analysis in relation to age

There were 7 children in the first age group (3 girls), 28 in the second (9 girls), and 65 in the third (39 girls). A summary of clinical characteristics, results of HRV analyses and statistical comparisons by the Kruskal-Wallis test are shown in Table 2. The results of the *post-hoc* analysis with trend analysis between age groups by the Jonckheere-Terpstra trend test are shown in Table 3 (only for parameters with a significant *p*-value in the Kruskal-Wallis test).

**Table 2:** **Clinical characteristics and results of HRV analysis of studied healthy children in three age groups with results of the Kruskal-Wallis test**.

	**1st age group 3-6 years old** ***N*** = **7**					**2nd age group 7–12 years old N** = **28**					**3rd age group 13–18 years old N** = **65**					**K-W test *p*-value**
	**Mean**	***SD***	**Median**	**25th p**.	**75th p**.	**Mean**	***SD***	**Median**	**25th p**.	**75th p**.	**Mean**	***SD***	**Median**	**25**	**75 P**	
Age [years]	4.4	1.3	4.0	3.3	5.8	9.6	1.7	10.0	8.0	11.0	15.5	1.6	15.0	14.8	17.0	
PQ [ms]	118.9	8.7	120.0	110.5	127.5	127.9	18.0	128.0	114.0	140.0	131.2	15.7	130.0	120.0	140.0	0.1120
QRS [ms]	77.0	13.0	80.0	65.5	87.5	83.3	8.1	81.0	79.0	90.0	87.3	8.1	86.0	80.0	90.0	0.0270
QTc [ms]	388.7	22.2	388.0	373.8	395.0	391.8	16.4	393.0	381.0	401.5	393.9	21.2	395.0	379.5	410.5	0.6176
Supraventricular beats in 24-h ECG [%]	0	0	0	0	0	0.8	2.8	0	0	0	1.1	5.4	0	0	0	0.5739
Ventricular beats in 24-h ECG [%]	0.4	1.1	0	0	0	0.2	1.0	0	0	0	1.5	4.4	0	0	0	0.5274
Duration of ECG recording [min]	1312.3	44.1	1327.5	1277.2	1345.6	1311	101.6	1306.1	1235.4	1419.2	1293.8	108.6	1293.8	1223.2	1380.3	0.8001
Mean RR [ms]	643.2	40.1	656.7	621.0	668.8	701.1	73.2	705.8	637.1	756.8	767.9	70.8	770.1	714.1	812.8	0.0000
SDNN [ms]	125.5	34.3	126.3	93.7	147.8	148.0	42.4	139.3	118.0	177.9	166.6	39.3	165.6	139.6	192.6	0.0111
ULF [ms^2^]	11638.2	7240.5	7617.0	6955.2	16066.3	16741.7	10074.6	13420.9	9735.0	21376.7	23528.6	11732.1	23091.7	14798.3	31390.6	0.0006
VLF [ms^2^]	1982.0	1172.1	1891.0	1102.1	2739.2	3169.6	1730.7	2475.7	1870.1	4152.9	4129.6	1984.3	3919.1	2787.9	5191.7	0.0025
LF [ms^2^]	1171.9	682.4	1213.8	717.6	1358.3	1809.3	1022.8	1558.6	1048.6	2444.5	2186.7	1174.9	1977.6	1338.6	2839.3	0.0304
HF [ms^2^]	1646.0	1462.8	1537.1	548.0	2252.4	2258.1	2563.8	1372.1	734.8	2726.5	1772.9	1606.2	1476.4	676.7	2058.0	0.8695
LF/HF []	1.0	0.6	0.8	0.6	1.1	1.2	0.6	1.1	0.9	1.5	1.6	0.7	1.6	1.2	2.2	0.0031
SD1 [ms]	51.7	27.4	59.5	25.4	67.2	51.0	29.1	45.1	28.6	66.2	47.8	22.4	44.5	32.3	55.9	0.9314
SD2 [ms]	168.7	44.6	168.4	129.3	195.5	201.3	55.5	191.0	162.3	238.9	229.2	53.8	230.4	190.7	267.5	0.0039
CL [%]	91.4	6.7	92.8	89.4	96.3	94.0	4.6	95.5	92.4	97.2	95.5	3.7	96.6	94.6	97.7	0.0228

**Table 3:** **Results of the *post-hoc* comparison of clinical characteristics and results of HRV analysis between age groups (only *p* < 0.05), and the Jonckheere-Terpstra trend test for the trend analysis along age groups**.

	**1st vs. 2nd age group**	**1st vs. 3rd age group**	**2nd vs. 3rd age group**	**J-T trend test**
Age	*p* < 0.05	*p* < 0.05	*p* < 0.05	< 0.00001
QRS		*p* < 0.05	*p* < 0.05	0.00836
Mean RR	*p* < 0.05	*p* < 0.05	*p* < 0.05	< 0.00001
SDNN		*p* < 0.05	*p* < 0.05	0.00340
ULF		*p* < 0.05	*p* < 0.05	0.00010
VLF		*p* < 0.05	*p* < 0.05	0.00090
LF		*p* < 0.05		0.01693
LF/HF		*p* < 0.05	*p* < 0.05	0.00073
SD2		*p* < 0.05	*p* < 0.05	0.00105
CL		*p* < 0.05		0.00721

The variance of the duration of PQ and QTc was not related to the age group. However, QRS duration significantly increased with age, and the trend for this increase was statistically significant. Although there was no significant relationship between the average duration of ECG Holter recording along age groups, the number of beats rejected from analysis was higher in older children than in the youngest. Nevertheless, there was no significant trend in the number of rejected beats with increasing age.

For HRV, with the exception of HF power and SD1, all other HRV parameters were significantly different between age groups. The duration of mean RR and values of SDNN, ULF, VLF, LF, LF/HF, and SD2 significantly increased while the relative contribution of long-term HRV to total HRV (CL) significantly decreased across age groups.

**Figure 1:**
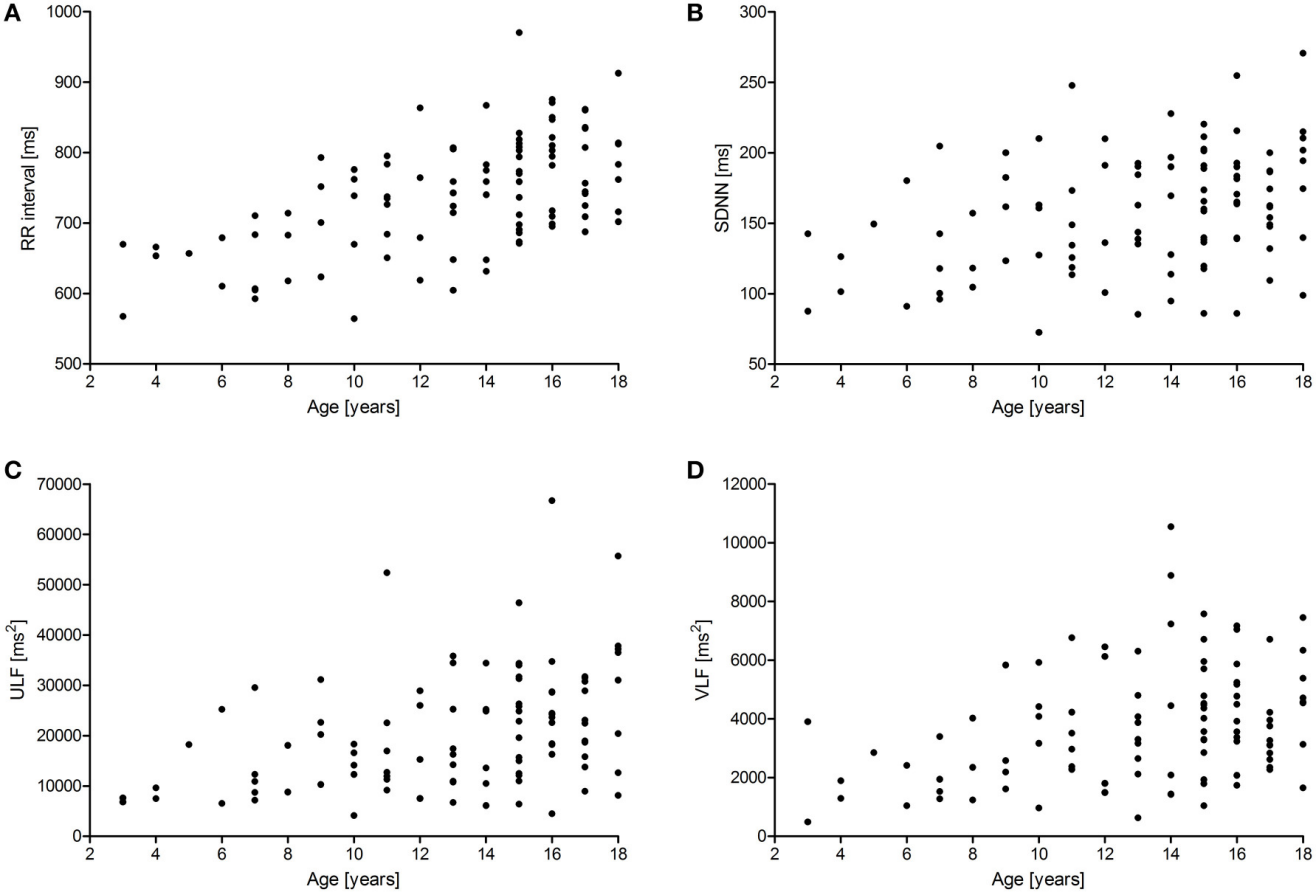
**Scattergrams showing relations between children's age and mean RR interval (A)**, SDNN **(B)**, the power of ULF **(C)**, and VLF **(D)**. For abbreviations, please refer to the main text.

**Figure 2:**
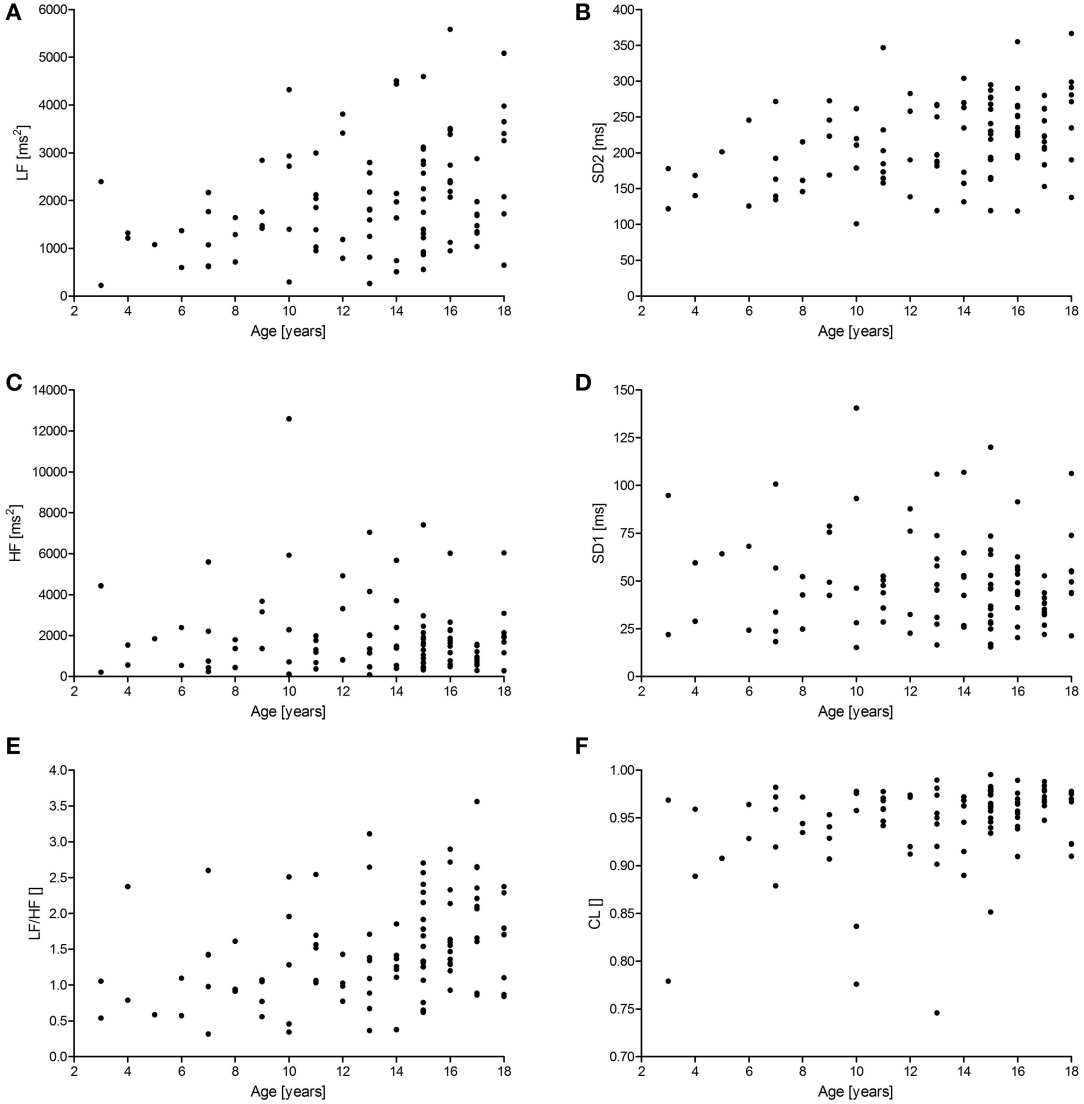
**Scattergrams showing relations between children's age and the power of LF (A)** and HF **(C)**, SD2 **(B)**, SD1 **(D)**, LF/HF **(E)**, and CL **(F)**. For abbreviations, please refer to the main text.

Figures 1, 2 show scattergrams for the relations between analyzed HRV parameters and children's age. The results of non-parametric correlation analysis between the age of healthy children and the studied HRV parameters are shown in Table 4. There was no significant correlation between the age of children and the power of HF or value of SD1. There was a significant and positive correlation between the age and the duration of mean RR interval, SDNN, ULF, VLF, LF, LF/HF, SD2, and CL. Out of all significant correlations, the strongest correlation was between the age and mean RR interval, and ULF; however, the strength of these correlations was at most moderate. The remaining significant correlations were rather weak. As shown in Figures 1 and 2, the analyzed relations between HRV and age appear to be non-linear.

**Table 4:** **Results of Spearman correlation (rho co-efficient) between the age of healthy children and studied HRV parameters from the 24-h ECG Holter recordings**.

	**Rho**	***P*-value**
Mean RR	0.55	< 0.0001
SDNN	0.33	0.0008
ULF	0.42	< 0.0001
VLF	0.35	0.0004
LF	0.30	0.0028
HF	−0.02	0.8758
LF/HF	0.38	0.0001
SD1	−0.04	0.6992
SD2	0.37	0.0002
CL	0.32	0.0012

## Discussion

With this study we applied the Poincare plot analysis and the Lomb-Scargle periodograms to the RR interval time series derived from the 24-h ECGs. The application of both methods for HRV to the 24-h ECGs in healthy children is one example of novelty brought by our study. Another novelty is the use of an index CL derived from the Poincare plots analysis of RR intervals which helps to estimate what is the relative contribution of the long-term variability to the total HRV at the cost of the short-term variability. Additionally, we provide reference values for these methods and a set of parameters derived from them—this is important as we carefully evaluated the health status of the enrolled children, used the recommended approach to HRV analysis (detailed ECG analysis with identification of beat types and using RR intervals only of sinus origin, high sampling rate of the ECG signal, and 24-h duration of these recordings). We have also found that the sex of healthy children does not contribute to the applied HRV indices. For the age, however, we found a statistically significant relationship with the values of most measured HRV parameters. It appears that as a child advances in age in the range of 3–18 years, most HRV measures (ULF, VLF, LF, LF/HF, SDNN, SD2, CL) increase. The only HRV indices which do not alter significantly with age are those reflecting fast-oscillations (HF) or short-term (SD1) variability of RR intervals, both of which correspond to HRV phenomena on short time scales.

### Clinical studies on HRV in children

There is a substantial number of reports on the clinical use of HRV in children, including newborn babies and infants. Reduced HRV, i.e., total power, VLF, LF, and HF, was observed in a group of 112 children with Fontan circulation compared with 66 control subjects (Dahlqvist et al., 2012). Massin and von Bernuth (1998) found reduced HRV in children with congenital heart disease, and that HRV was lower in patients with more advanced heart failure assessed by the NYHA functional class. Dias de De Carvalho et al. (2014) observed that children with the Attention Deficit Hyperactivity Disorder (ADHD) had increased HRV indices describing the parasympathetic activity (i.e., HF, rMSSD or percentage of differences between adjacent normal-to-normal intervals greater than 50 ms—pNN50) compared with control group. Kardelen et al. (2006) reported that HRV parameters were markedly reduced in children with type 1 diabetes compared with healthy children. Akinci et al. (1993) found using 24-h ECG Holter recordings that diabetic children with poor glycaemic control had significantly reduced HRV indices compared with healthy children (for example LF and HF). Soares-Miranda et al. (2011) noticed that HF is reduced in girls with central fat above the median value compared with girls with lower amount of central fat. Spassov et al. (1994) analyzed the influence of the retardation of intrauterine growth on heart rate and HRV, and reported that small-for-gestational age newborn babies had shorter RR interval and reduced HRV in comparison with the appropriate-for-gestational age newborns during sleep. Schechtman et al. (1989) found decreased HRV in the long-term recordings obtained from infants under 1 month who later succumb to sudden infant death syndrome (SIDS). However, Antila et al. (1990) did not observe any significant differences in HRV between 17 infants with SIDS and 23 healthy infants. These examples clearly show that there is a lot of interest in HRV in children in a variety of clinical conditions.

### Physiological studies on HRV in children

#### Sex of children and HRV

The issue of the children's sex and its relation to HRV was a subject of several studies. Silvetti et al. (2001) studied time domain HRV parameters in 24-h ECGs in 103 healthy children starting from one-year-old babies up to adolescents and found that neither SDNN, pNN50 nor rMSSD were related to gender. Rękawek et al. (2003) carefully investigated a group of 372 children aged from 4 to 18 years and measured HRV by many time domain and spectral HRV parameters in the 5- and 20-min ECG segments selected from the day and night of 24-h ECG Holter recordings. In their comments Rekawek et al. report that sex was the least significant contributor to time domain HRV and the most significant to the total power of HRV—unfortunately the authors do not present detailed data for this type of analysis, and by studying the tables with the summary of the results the reader can guess that the sex effects on HRV were either missing or very week. Seppälä et al. (2014) examined 465 mainly prepubertal children in a very narrow age range of 6–8 years. HRV was analyzed in the resting ECGs of 1- and 5-min duration, and the authors did not find any significant difference between girls and boys in the studied HRV parameters. Umetani et al. (1998) studied time domain HRV parameters in 24-h ECGs in a group 260 healthy subjects in a wide range of age between 10 and 99 years old. In this study, there were only 30 children up to 19 years old, however for the analysis of sex effects, these children were combined with adults up to 29 years old—in summary, there were significant differences for the time domain parameters such as SDNN, SDANN (standard deviation of 5-min means of RR intervals), rMSSD or pNN50 between combined groups containing 32 boys and young men vs. 40 girls and young women—no separate analysis of sex differences between the studied children were shown. Goto et al. (1997) investigated 25 healthy boys and 35 healthy girls between 3 and 15 years old and found no significant differences by sex in any of the studied HRV indices, neither in time nor in frequency domains. Michels et al. (2013) studied a group of 460 children in the age between 5 and 10 years old. The children were selected by random cluster sampling from the region of Aalter in Belgium, and RR intervals were derived not from an ECG but from the elastic electrode belt Polar Wearlink (Polar, Finland) placed around the chest. For the HRV, both time and frequency domain parameters were studied in 5-min segments of RR intervals. Mean RR interval, SDNN, rMSSD, pNN50, the powers of VLF, LF, and HF were significantly larger in boys than in girls, but there was no significant difference in any of the relative measures of spectral HRV indices (LF/HF, LFnu, HFnu). Fukuba et al. (2009) examined 48 boys between 8 and 14 years old and 88 girls between 8 and 20 years old who were asked to breathe periodically at a rate of 15 breaths/min. For the HRV analysis, the authors used 3-min ECGs and measured the rate of HF to TP as an index of parasympathetic, and LF/HF as an index of sympathetic control of heart rate—they found no sex differences in these indices. Sharma et al. (2015) conducted a study in a group of 250 boys and 189 girls in the age between 12 and 17 years old who were then divided into non-athletes and athletes according to the definition that an athlete had represented the school at state, national or international level athletic interscholastic sport event and was undergoing supervised physical training. The time and spectral domain HRV was computed in RR intervals gathered with the use of the Bioharness sensor placed on a chest strap (Zephyr, USA) in 5-min resting recordings. Among the non-athletes, girls had significantly higher values of rMSSD, NN50, TP, LF, and HF than boys, and there were no significant sex differences for SDNN and LF/HF (and logically LFnu and HFnu). However, among athlete adolescents, girls presented significantly higher SDNN than boys, and there were no other significant differences among other HRV indices between sexes. Jarrin et al. (2015) investigated a large group of 1,036 children (481 boys and 555 girls) in a very narrow age range 9–11 years (mean 10 years old). The authors found many statistically significant differences (p at least < 0.01) suggesting that boys, compared to girls, have significantly higher values of time and spectral HRV parameters with the exception of LF/HF which appeared to be lower. However, the analysis of mean values of these parameters and their standard deviations show that the differences in the means were rather small, for example the comparison of boys vs. girls for SDNN shows 89.4 ± 26.1 vs. 84.4 ± 23.1 ms, and for LF 1,587.7 ± 1,040.1 vs. 1,411.2 ± 934.4 ms^2^, respectively. The most curious comparison is revealed for LF/HF where it is 2.1 ± 0.8 for boys, and 2.2 ± 0.9 for girls. Galeev et al. (2002) examined probably the largest group of 5,400 children in the age between 6 and 16 years old for the purposes of HRV study, and finally selected 300 children for each year of age collecting together 3,300 sets of RR intervals. For the spectral HRV analysis, 128 consecutive RR intervals were selected whether for the time domain HRV 2000 consecutive RR intervals were chosen. SDNN was significantly higher in boys than in girls for 12, 13, and 14 years old children. TP was significantly higher in boys in the age interval 11–15 years old, VLF was larger in boys in the age 13–15 years but lower for 12 years old. Sex differences in LF and HF were present for children in the age between 15 years but in changing patterns, i.e., once these parameters were higher in boys, then in girls, and then again in boys. The LF/HF was larger in boys than in girls between 12 and 15 years old. For children at age of 16 years there were no sex differences in any of the HRV studied parameter. Such studies as Jarrin et al. (2015) and Galeev et al. (2002) raise a question whether all statistically significant differences are clinically relevant? Whether, in fact, the small differences in the values of HRV measures are statistically different because of the mass effect, i.e., large sample sizes influencing *p*-value (Farland et al., 2016). Gasior et al. (2015) recorded a resting ECG in 158 boys and 173 girls in age between 6 and 13 years old and selected 5-min segments for the time and spectral HRV analysis. During their analysis the authors manually corrected the erroneous beats by removing one RR interval before and one after each non-sinus beat, and then replacing the missing RR intervals by mathematically interpolated RR intervals. Compared with girls, boys presented significantly higher HRV values (SDNN, rMSSD, pNN50, TP, LF, and HF) but lower heart rate. However, in the linear multiple regression analysis adjusted for sex, all HRV parameters were significantly related to heart rate but not sex of the children—this suggests that if there are any sex differences in HRV in children they are rather an effect of heart rate than the sex itself. Cysarz et al. (2011) collected up to 24-h ECG Holter recordings in 409 healthy children, adolescents and young adults in the age range between 1 and 22 years. In this group there were 220 girls or women and 189 boys or men. In addition to a set of parameters assessing the non-linear measures for irregularity and fractal properties of RR intervals, they computed SDNN and ULF for the whole 24-h ECGs and, additionally, VLF, LF, HF, and LF/HF for each consecutive 1-h epoch of the recording. These 1-h epochs for VLF, LF, HF, and LF/HF were then averaged separately for daytime (from 9 a.m. to 6 p.m) and night-time (from 0 a.m. to 6 a.m.). Both SDNN and all spectral measures of HRV were significantly lower in girls and young women than in boys and young men. With our study we observed no differences in HRV parameters (neither from the spectral analysis by Lomb-Scargle method nor Poincare plots) calculated in the 24-h ECGs between healthy girls and boys. Additionally, we did not find any influence of the interaction between age and children's gender on the studied HRV parameters. In general, it may be concluded that in contrast to adults, gender seems to have no influence on HRV in children, at least with the methods we used and for the 24-h ECGs. In summary of the gender relation to HRV, the available data are sparse and contradicting. Some authors show that boys have significantly higher HRV, some other show the reverse effects whereas some, including us, reveal no association of HRV with the sex of children.

### Age of children and HRV

There are numbers of studies investigating the relationship between the age of healthy children and HRV. Clairambault et al. (1992) studied 24 healthy sleeping newborns who were born between 31 and 41 weeks of the conceptional age. They found that HRV measured by spectral analysis was increased from the premature (31–36 weeks) to the intermediary (37–38 weeks) for both HF and LF, and to the full term (39–41 weeks) of the conceptual age for the LF. Silvetti et al. (2001), who investigated time domain HRV, found that there was a decrease of mean RR interval and SDANN with an increasing age of children (between the age of one year and adolescence), but no relation was observed with other HRV parameters. Goto et al. (1997) examined 60 healthy children aged 3–15 years for HRV. Time domain HRV measures showed a significant increase in SDNN in the group 3–9 years old and a decline in children 9–15 years old. For the spectral HRV assessment, 10-min recordings were used—the authors showed that HF and LF increased in children 3–6 years old and then gradually declined in children who were 6–15 years old. The LF/HF ratio increased in the group of 3–6 year old and then no significant differences were observed in older children up to 15 years old. Rękawek et al. (2003) investigated healthy children between 4 and 18 years old and found a significant correlation between several time domain HRV parameters (e.g., SDNN or SDANN) and age. However, similar correlation with age was absent for both rMSSD and pNN50. For the spectral HRV for selected 20-min ECGs from the day and night only some of these parameters (e.g., VLF, LF, HF, and LF/HF) were significantly correlated with age. Michels et al. (2013) in their study of children between 5 and 10 years old found that age correlated with both time and frequency domain parameters in boys and girls. Fukuba et al. (2009) did not find any significant correlation between age and neither the parasympathetic (HF/TP) nor sympathetic (LF/HF) indices of the control of heart rate. Although Sharma et al. (2015) provided reference data for many HRV parameters for children in the age between 12 and 17 years old, they did not report any association between age and the studied parameters. Gasior et al. (2015) did not find any significant correlation between age (children in the range 6–13 years old) and HRV parameters derived from time or frequency domains. Lenard et al. (2004) analyzed time domain and spectral HRV in 10-min resting ECGs in 137 healthy children and young adults divided into 4 age groups, i.e., 7–10 years old, 11–14 years old, 15–18 years old, and 19–22 years old. They found that only SDNN, LF, and HF were significantly larger in the oldest children 15–18 years old compared to the middle group between 11 and 14 years old. Finley and Nugent (1995) investigated 61 healthy children and young adults from 1 month to 24 years old using 24-h Holter ECG recordings. From these recordings segments of 1-h duration for the awake state, quiet and active sleep were chosen for the computation of spectral HRV indices. The power of LF and LF/HF changed significantly with age during both quiet and active sleep whereas HF only changed during quiet sleep. The total power was not related to age irrespective of the sleep or awareness state in this study. Massin and von Bernuth (1998) recorded 24-h ECGs in 210 children aged between 3 days to 14 years old to analyse time and frequency domain HRV indices, including SDNN, SDANN, rMSSD, pNN50, VLF, LF, HF, and LF/HF. They noticed that age was significantly and positively correlated with all studied HRV parameters. Galeev et al. (2002) who examined altogether 3,300 sets of RR intervals from children found that practically all of time and spectral HRV indices changed more or less with age, usually in a waveform. Compared with younger children, TP, VLF, and LF started to differ from the age of 11 while SDNN, rMSSD, and HF from the age of 12. In their study, Cysarz et al. (2011) found age related variation in the SDNN, ULF, VLF, LF, and LF/HF—the value of these parameters was, in general, increasing with age. In contrast, there was no relation between HF and age of the studied children and young adults.

In our study we also observed a series of significant correlations between age and most time domain, spectral and Poincare plot HRV parameters computed in the 24-h ECGs. Although the magnitude of these associations was rather modest to medium, most values of the HRV parameters increased significantly with age in the range of 3–18 years. This is true for the measures of total (SDNN), ultra low (ULF), very low (VLF) and low frequency (LF) as well as long-term (SD2) variability—all of these parameters are different parts of the total variance (i.e., squared SDNN).

Our observations on the relation of age with at least some of the HRV measures, i.e., SDNN and LF, are in concordance with the studies by other authors (Finley and Nugent, 1995; Goto et al., 1997; Massin and von Bernuth, 1998; Galeev et al., 2002; Rękawek et al., 2003; Lenard et al., 2004; Cysarz et al., 2011; Michels et al., 2013). However, it appears that not all HRV indices that contribute to total HRV show such a significant relationship with age. In our case, neither HF nor SD1 alter significantly with age. This observation is partially similar to the study of Silvetti et al., Rekawek et al., Gasior et al., and Cysarz et al. Silvetti et al. (2001) who reported that two measures of short-term variability, i.e., pNN50 and rMSSD (which is the rescaled SD1 and thus identical in interpretation with it) were only partially related to age—these are correlated with age only for children up to 10 years old—later, this relationship is no longer significant. Rękawek et al. (2003) did not find such correlation with either RMSSD or pNN50 in their group. In spectral analysis, there were significant correlations between age and HF both for the 20-min ECGs from day and night, however these correlations were very weak (r coefficient −0.13 and −0.11, respectively). In the study by Gasior et al. (2015) no significant correlation between children's age and neither rMSSD nor HF were observed. Cysarz et al. (2011) found that HF was constant in children up to 13 years old and then slightly declined but in general it was not correlated with age. Although both HF and rMSSD (or SD1) come from different methods, in general they describe similar physiological phenomena of short-term or high frequency HRV (Task Force of the European Society of Cardiology the North American Society of Pacing Electrophysiology, 1996; Brennan et al., 2001; Guzik et al., 2007, 2010; Heathers, 2014; Sanchez-Gonzalez et al., 2015; Sassi et al., 2015), and according to the previous reports and our findings, we may conclude that the association between age and the measures of fast oscillations influences on HRV are either weakly or not at all correlated with the age of healthy children, at least when measured in 24-h ECGs by Poincare plots or Lomb-Scargle periodograms.

There are several differences between our study and the other investigations on HRV in healthy children. In a variety of studies, ECGs of different duration were used. This duration ranged from 1 min (Seppälä et al., 2014), through 3 (Fukuba et al., 2009), 5 (Rękawek et al., 2003; Michels et al., 2013; Seppälä et al., 2014; Gasior et al., 2015; Sharma et al., 2015), 10 (Goto et al., 1997; Jarrin et al., 2015), 20 (Rękawek et al., 2003) min to 5 (Goto et al., 1997), 6 for the night-time and 9 for the daytime (Cysarz et al., 2011), and 24 h (Umetani et al., 1998; Silvetti et al., 2001; Cysarz et al., 2011; our study). Galeev et al. used an unusual approach by analysing RR intervals of exactly 128 consecutive RR intervals for spectral HRV or at least 2000 RR intervals for time domain HRV (Galeev et al., 2002). Usually many different computational methods (time domain, spectral analysis by Fast Fourier Transform or Lomb-Scargle periodograms, and Poincare plots of RR intervals) were applied for HRV analysis. We used ECG Holter recorders with a sampling frequency of 1,000 Hz whereas in older studies it was lower, usually 128 Hz or sometimes 500 Hz. Higher sampling frequency increases the accuracy of RR intervals and thus HRV analysis (Piskorski and Guzik, 2011). Similarly to Laguna et al. (1998), for spectral HRV analysis we applied the Lomb-Scargle periodograms of RR intervals while other studies used Fast Fourier Transform (FFT), autoregressive models or wavelet decomposition analysis (Moody, 1993; Task Force of the European Society of Cardiology the North American Society of Pacing Electrophysiology, 1996; Verlinde et al., 2001; Pichon et al., 2006; Rajendra Acharya et al., 2006; Cysarz et al., 2011; Heathers, 2014). The FFT requires that the signal is sampled at equal intervals and since this condition is broken in RR intervals, the signal needs to be resampled. In contrast, the Lomb-Scargle periodograms of the RR intervals approach does not have similar requirements and limitations (Lomb, 1976; Moody, 1993; Piskorski et al., 2010). It can use unequally sampled signals such a time series of RR intervals which is naturally irregular. According to the recommendation of Moody (1993) and studies by Laguna et al. (1998), the Lomb-Scargle periodograms are the method of choice for the spectral HRV analysis as this method is better suited for this purpose than FFT. Laguna et al. (1998) goes even further after studying the effects of uneven sampling and the spectrum obtained by FFT and the Lomb-Scargle method, and suggest that the Lomb-Scargle approach is superior to FFT. Most of the times both methods for spectral HRV will give similar results, but in cases of some unforeseen violations of the assumptions of the methods, the Lomb-Scargle periodogram is closer to reality than FFT. We are, however, aware that the Lomb-Scargle periodogram analysis of RR intervals has also some limitations which we mentioned earlier. One of such limitations is the loss of the information about phase in the analyzed signal, however this information is uninterpretable in case of spectral analysis of RR intervals and thus not used at all.

To the best of our knowledge, this study is the first report on the application of the Lomb-Scargle periodograms method to 24-h ECG Holter recordings in healthy children, and for this reason these results can be used as reference values by other authors. It also appears that we are the first to use Poincare plot analysis of RR intervals for HRV derived from 24-h Holter ECG recordings in this group. In addition, we also used a new parameter—CL—quantifying the relative contribution of the long-term HRV to total HRV. This parameter helps to understand what proportion of total HRV derives from the long-term HRV—the remaining amount comes from the short-term HRV. Since there is a positive relationship between the age of children and CL, it follows that with the process of growing up there is an accumulation of long-term effects at the cost of the short-term input to the total HRV. This might be caused by respiratory sinus arrhythmia and its gradually weakening effects on the heart rate and HRV with advancing age of children, and that other physiological effects start having stronger influences on HRV. On the other hand, CL might also be interpreted as a relative index reflecting the expression of respiratory sinus arrhythmia on HRV.

### Limitations of the study

Some limitations of our study must be recognized. First, we used HRV, which is an indirect measure of the cardiac autonomic control. However, HRV is commonly applied for this purpose in a number of studies and even recommended for this purpose in specific guidelines (Task Force of the European Society of Cardiology the North American Society of Pacing Electrophysiology, 1996; Sassi et al., 2015)—one of the most beneficial features of this approach is a complete lack of invasiveness of HRV measurement. Second, we studied only 100 healthy children. The reason was that selection process was very detailed, careful and required a lot of effort to select such children from over 1,600 patients visiting the Outpatient Cardiac Paediatric Clinic who underwent a detailed clinical evaluation including echocardiography and 24-h ECGs recording. We took care of the comfort of all studied children as well as data quality. During this process we did not use special forms or clinical/research tools designed for specific purposes like the Growing and Changing Questionnaire for the evaluation of maturity, the Eurofit fitness test battery or maximal oxygen consumption (VO2max) for the physical fitness or total body impedance for the body composition analysis (Michels et al., 2013; Jarrin et al., 2015; Sharma et al., 2015)—looking at the possible associations between HRV measures and many potential covariates collected in the enrolled children was not the aim of our research. To get the best possible results we also applied a specific method for spectral HRV analysis, which is the Lomb-Scargle periodogram and that is recommended for the HRV analysis (Moody, 1993). Third, we also provide some completely new data, e.g., from Poincare plots and Lomb-Scargle periodograms analysis or with a new HRV measure, i.e., CL.

## Conclusions and potential implications

Based on previous studies which differ in so many aspects with our study it is difficult to clearly draw general conclusions on the relation of the HRV derived from 24-h ECGs to children's age and sex. For these reasons, we may make conclusions based only on our findings.

We did not find any gender differences in many HRV parameters computed for the 24-h ECGs by the Poincare plots and Lomb-Scargle periodograms in healthy children. However, we found that there is a significant correlation between age and the expression of most of HRV parameters which represent total HRV or its contributors from the long-term or ultra-low, very low and low frequency oscillations. We could not observe similar associations between age and the HRV contributors derived from the short-term effects or high frequency oscillations. The effects of age on the tested HRV indices was not modified by children's sex.

The potential implication of our study is using its results as reference values for the Poincare plots and Lomb-Scargle periodograms analyses in HRV studies in children, both in physiological and clinical investigations. Another implication comes from the interpretation of the CL parameters –it appears that with increasing maturity the contribution of long-time variability to the total HRV increases at the cost of the short-term variability in healthy children.

## Ethics statements

Poznan University of Medical Science Bioethical Committee Parents of all children as well as children of at least 7 years old themselves gave their informed consent for the enrollment to the study. All tests were non-invasive, painless and were additive to the standard examination.

## Author contributions

MS, KG, and WB: substantial contributions to the selection of children, their clinical evaluation, acquisition of Holter ECGs and their analysis, interpretation of data for the work; TK, JP, and PG: Signal analysis, computation of HRV parameters, data interpretation. WB, JP, AW, and PG: the conception or design of the work, drafting the work or revising it critically for important intellectual content, final approval of the version to be published, work on corrections of the manuscript and replies to reviewers comments; WB: Agreement to be accountable for all aspects of the work in ensuring that questions related to the accuracy or integrity of any part of the work are appropriately investigated and resolved. PG: corresponding author.

### Conflict of interest statement

The authors declare that the research was conducted in the absence of any commercial or financial relationships that could be construed as a potential conflict of interest.
